# Understanding the impact of ER stress on lung physiology

**DOI:** 10.3389/fcell.2024.1466997

**Published:** 2024-12-18

**Authors:** Zhiling Fu, Wei Wang, Yuan Gao

**Affiliations:** ^1^ Department of Anesthesiology, Shengjing Hospital of China Medical University, Shenyang, Liaoning, China; ^2^ Department of Pulmonary and Critical Care Medicine, Shengjing Hospital of China Medical University, Shenyang, Liaoning, China

**Keywords:** ERstress, upr, autophagy, aging, lung dysfunction

## Abstract

Human lungs consist of a distinctive array of cell types, which are subjected to persistent challenges from chemical, mechanical, biological, immunological, and xenobiotic stress throughout life. The disruption of endoplasmic reticulum (ER) homeostatic function, triggered by various factors, can induce ER stress. To overcome the elevated ER stress, an adaptive mechanism known as the unfolded protein response (UPR) is activated in cells. However, persistent ER stress and maladaptive UPR can lead to defects in proteostasis at the cellular level and are typical features of the lung aging. The aging lung and associated lung diseases exhibit signs of ER stress-related disruption in cellular homeostasis. Dysfunction resulting from ER stress and maladaptive UPR can compromise various cellular and molecular processes associated with aging. Hence, comprehending the mechanisms of ER stress and UPR components implicated in aging and associated lung diseases could enable to develop appropriate therapeutic strategies for the vulnerable population.

## 1 Introduction

The process of aging is intricate, involving the gradual decline of tissues and organs throughout the body, representing a standalone risk factor for various age-related illnesses (Zhang W. et al., 2020). As life expectancy rises and the aging demographic expands globally, it is crucial from a public health standpoint to comprehend the mechanisms by which aging contributes to an escalating vulnerability to chronic illnesses and disabilities over time (Ferrucci and Fabbri, 2018).

The human lungs, constituting most of the body’s surface area, serve as a distinctive interface for engaging with the external environment and continually face biological, chemical, mechanical, and immunological stress throughout an individual’s lifespan (Schneider et al., 2021). As age progresses, the lungs undergo a gradual weakening characterized by structural changes that hinder gas exchange and weaken defensive mechanisms. Consequently, this susceptibility heightens the risk of lung injuries induced by environmental exposures (Sharma and Goodwin, 2006; Bowdish, 2019) and contributing to increased susceptibility to several lung diseases. Notably, conditions like chronic obstructive pulmonary disease (COPD) and idiopathic pulmonary fibrosis (IPF), prevalent in the elderly, not only align with accelerated aging but also replicate the aged lung’s structural and physiological traits (Ito and Barnes, 2009; Pardo and Selman, 2016). The COVID-19 pandemic has highlighted the heightened vulnerability of the elderly to acute respiratory distress syndrome (ARDS) (Chen et al., 2021). Additionally, while asthma is traditionally viewed as a childhood ailment, severe forms with elevated morbidity and mortality rates are more prevalent in the elderly population (Gillman and Douglass, 2012).

Recent findings suggest that intervening in age-related degenerative biological processes can mitigate or postpone the emergence of various age-related diseases. Experimental trials in model animals have further established a fact regarding aging that it is a manageable as well as alterable condition (de Cabo and Mattson, 2019; Abbasi, 2017).

The endoplasmic reticulum (ER) is a membranous network consisting of branching tubules and flattened sacs. It plays a crucial role in overseeing the synthesis, folding, and processing of over one-third of all cellular proteins (Walter and Ron, 2011). Within cells equipped with an endoplasmic reticulum (ER), such as those found in the lungs, the secretory capacity undergoes constant influence from both physiological demands and pathological disturbances. This results in a disruption of ER homeostasis, recognized as ER stress (Dastghaib et al., 2021). In response to heightened ER stress, adaptive mechanisms, collectively termed the unfolded protein response (UPR), come into play to restore equilibrium (Wiseman et al., 2022).

The aging process has a significant impact on cellular functions, mainly affecting the chaperone system, leading to an essential role in heightened protein misfolding and aggregation (Macario and Conway de Macario, 2002). Contemporary findings propose an association between disturbances in proteome homeostasis (proteostasis) and the standard aging process, contributing to age-related disroders in lungs (Schneider et al., 2021; Balch et al., 2014). Consequently, it is unsurprising that the aging lung exhibits various indications of disruption in cellular homeostasis due to ER stress. Additionally, malfunctioning of cellular homeostasis due to ER stress can impair other metabolic functions responsible for aging. Therefore, understanding these inter-relationships is important because interventions targeting these relationships should have a beneficial effect on delaying aging and preventing aging-related disorders. In this context, we examined ER stress dynamics for aging and associated lung disorders and discussed attempts to target ER stress in the lungs to achieve an antiaging effect; this research could enable to develop therapeutic strategies for the vulnerable population.

## 2 Overview of ER stress and UPR

The ER constitutes a complex structure of branching tubules and flattened sacs, overseeing the synthesis, folding, and processing of majority of cellular proteins (Hetz and Papa, 2018). The ER is also responsible for Ca^2+^ storage and biosynthesis of lipids and steroids (Hetz et al., 2020). In the ER, chaperones and enzymes ensure proper folding and modification of secretory proteins, maintaining biosynthetic function (Walter and Ron, 2011). Upon attaining the correct conformation, chaperones within the ER disengage from proteins, enabling their exit. Subsequently, cells efficiently remove improperly folded proteins through stringent quality control mechanisms such as ER-associated degradation (ERAD) and ER-phagy.

While the endoplasmic reticulum (ER) is resilient, cells frequently operate near their secretory capacity limits. Various factors, including environmental, genetic, disease-related, or aging-related influences, can perturb protein folding efficiency, resulting in the buildup of misfolded or unfolded proteins in the ER lumen, causing “ER stress” (Walter and Ron, 2011). In response to ER stress, the UPR signal transduction pathway is activated within the cell to safeguard the genuineness and integrity of proteins. The UPR modifies cellular transcription and translation programs, impacting diverse parameters related to protein metabolism, redox homeostasis, and apoptosis (Walter and Ron, 2011; Wang and Kaufman, 2012).

**FIGURE 1:**
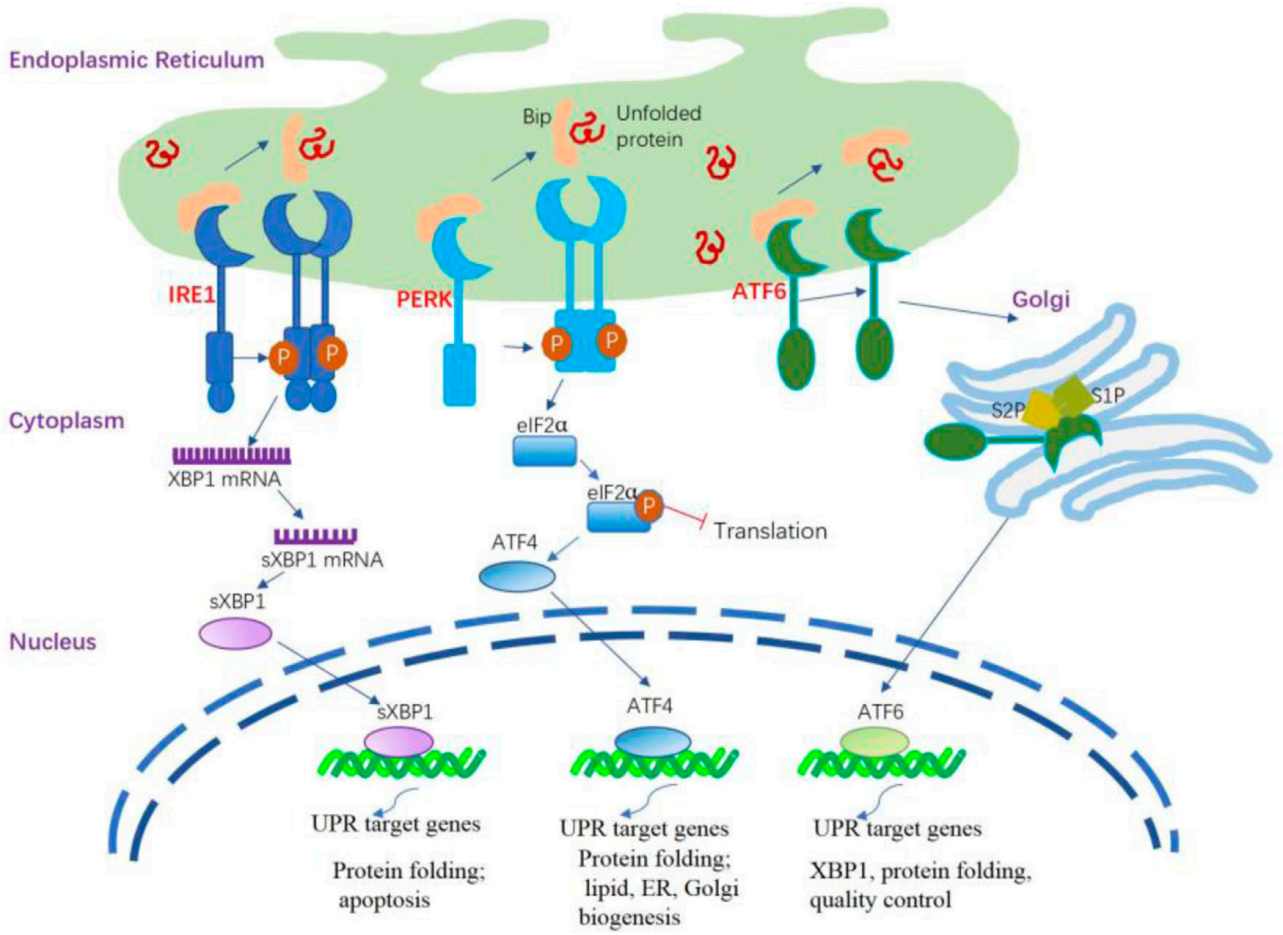
ER stress and activation of the UPR signaling pathways. The UPR is triggered by ER stress sensors, namely IRE1, PERK, and ATF6 upon UPR target genes related dissociation of BiP. The activation of PERK through its dimerization and autophosphorylation suppresses the overall protein synthesis by eukaryotic translation initiation factor 2 subunit α (eIF2α) phosphorylation. PERK activation also increases the formation of the transcription factor ATF4 that regulates several components involved in amino acid transport and protein folding. IRE1α is activated following its dissociation from BiP and spontaneously undergoes homodimerization and autophosphorylation; the modified IRE1α induces splicing of the X-box binding protein 1 (XBP1) mRNA to generate sXBP1 that regulates several UPR target genes responsible for protein folding, lipid synthesis, and quality control of cellular proteins. The activated ATF6 is processed in the Golgi apparatus by site 1 protease (S1P) and site 2 protease (S2P) to release the cytosolic domain ATF6f that regulates some UPR target genes related to XBP1, protein folding, and quality control of cellular proteins.

Briefly, the UPR signal comprises three major stress-related proteins situated on the ER membrane: protein kinase R-like ER kinase (PERK), activating transcription factor 6 (ATF6), and inositol-requiring enzyme 1 (IRE1) (Wiseman et al., 2022) (Figure 1). PERK and IRE1 are type I transmembrane proteins that have a luminal domain for sensing unfolded proteins and a cytoplasmic kinase domain. In addition, IRE1 has a cytoplasmic endoribonuclease activity domain (Karagöz et al., 2017). In mammals IRE1 has two isoforms, namely the IRE1α and IRE1β. IRE1α is primarily expressed in almost all tissues. whereas IRE1β is found exclusively in intestinal epithelial and airway mucous cells (Akhter et al., 2020). IRE1β functions at the interface of epithelial cells with the external environment, maintaining mucosal homeostasis. ATF6 is a type II transmembrane protein that resides in the ER membrane. It has a luminal domain that senses ER stress and a cytoplasmic transcription factor domain (Lei et al., 2024).

The initial phase of the UPR commences with the activation of PERK. When unfolded proteins accumulate in the ER, they bind to BiP, causing BiP to dissociate from PERK. This dissociation enables PERK to oligomerize and autophosphorylate (Brown et al., 2014). This process inhibits overall protein synthesis by phosphorylating eIF2α, reducing mRNA translation and preventing protein buildup in the ER. Notably, it enhances the translation of specific mRNAs, including ATF4, which activates CHOP, illustrating the UPR’s cascade to maintain cellular homeostasis under ER stress (Han et al., 2013; Tabas and Ron, 2011). CHOP undergoes nuclear translocation, regulating elements of the B cell lymphoma protein 2 (BCL-2) protein family to initiate apoptosis mediated by ER stress. During ER stress, IRE1α activates after dissociating from GRP78/BiP, homodimerizes, and autophosphorylates. This leads to the activation of the cytosolic RNase domain, which splices the mRNA encoding XBP1 (X-box-binding protein 1), removing a 26-nucleotide intron. This splicing event converts unspliced XBP1 (uXBP1) into spliced XBP1 (sXBP1), a potent transcription factor (Calfon et al., 2002; Yoshida et al., 2001). sXBP1 functions as an active transcription factor, overseeing the regulation of various genes associated with protein functions as well as lipid, ER, and Golgi biogenesis (Acosta-Alvear et al., 2007; He et al., 2010). Under normal conditions, IRE1β restricts the activity of IRE1α and UPR signaling (Grey et al., 2020). During stressful situations, IRE1β can still play a role in XBP1 splicing, thereby helping to adjust the protein folding capacity of epithelial cells and restore mucosal homeostasis. Additionally, IRE1β has been identified as a negative regulator of IRE1α, as its overexpression is able to decrease IRE1α-dependent XBP1 splicing. As such, IRE1β is believed to alleviate ER stress in the mucosal epithelium during inflammation-induced diseases (Le Goupil et al., 2024). Upon encountering ER stress, ATF6 is activated through a mechanism distinct from both IRE1 and PERK. BiP separates from ATF6α, revealing the Golgi localization signal of ATF6α. ATF6 transitions to its monomeric form and moves to the Golgi apparatus. Processing in the Golgi via COP II vesicles, where S1P and S2P cleave ATF6 in the transmembrane domain, produces the active ATF6 fragment and releases the cytosolic domain of ATF6 (Ye et al., 2004; Chen et al., 2002). Similar to sXBP1, the ATF6 fragment actively medicates the gene regulation related to XBP1 and protein functions (Janssens et al., 2014).

Initially deemed protective, the UPR serves to decrease protein load and alleviate ER stress. Nevertheless, if the UPR persists in an extended or overly activated state, it can transition into a maladaptive mode, posing the risk of irreversible cell injury and, in severe cases, cell death. (Hetz and Papa, 2018).

## 3 ER stress and the UPR in lung aging

### 3.1 Aging of the lung

The aging of the lungs can ensue from the cumulative alterations in the cellular systems, reflecting the interplay between injury and repair within the lung (Abadie et al., 2005). These changes disrupt lung cell homeostasis by affecting proteostasis, cellular senescence, stem cell reservoirs, oxidative stress, and mitochondrial function.

The biological function of the lungs is compromised in advanced age, irrespective of the presence of specific diseases. The major lung components, including respiratory epithelium, lung progenitor cells, the interstitium, and pulmonary immune cells, are altered in the aged lung (Schneider et al., 2021), eventually leading to a reduced surface for gas exchange (Lalley, 2013), decreased mucociliary clearance in both upper and lower airways (Proença de Oliveira-Maul et al., 2013; Svartengren et al., 2005), and impaired repair and regenerative ability, subsequently resulting in the development of emphysema and pulmonary fibrosis (Kotton and Morrisey, 2014). Interactions between aging, senescence, and environmental factors lead to the breakdown of stress response pathways, contributing to the development of various lung diseases (Luppi et al., 2021).

The structural and functional changes in the aging lung collectively lead to decreased lung function, diminished compensatory mechanism, and enhanced susceptibility to pulmonary diseases. Consequently, lung diseases disproportionately affect elderly individuals. Therefore, to delay these aging-related lung diseases, it is important to modify the biological processes that deteriorate with age.

### 3.2 ER stress-related changes in the aging process

Aging can influence the structure of the ER inside the cell (Erickson et al., 2006; Moltedo et al., 2019). In individuals with COPD, lung fibroblasts exhibit a less organized and patchy ER structure compared to the reticulated ER structure observed in the lung fibroblasts of both never-smokers and ever-smokers (Weidner et al., 2018).

The functionality of the UPR is compromised as a result of the age-related gradual deterioration in the apparatus essential for the proper folding of proteins; consequently, in aged individuals, UPR activation cannot rescue ER stress (Naidoo, 2009). For example, salient chaperones and enzymes such as GRP78, PDI, calnexin, and GRP94, in the ER are impaired during the aging process (Nuss et al., 2008). This functional decline in chaperones and enzymes may be caused by progressive oxidation with advancing age (Nuss et al., 2008). Normally, the protective UPR and apoptotic signals are in a balanced state in a cell; however, this equilibrium might incline towards the proapoptotic condition as age progresses, attributable to the gradual deterioration in the UPR. Aging diminishes PERK expression and kinase activity, inhibiting the cytoprotective phosphorylation of eIF2α. This condition promotes protein translation and the expression of proapoptotic proteins in the ER during aging (Hussain and Ramaiah, 2007; Paz Gavilán et al., 2006). Similarly, CHOP and caspase-12 expression was induced in aged, stressed rats, but not in young, stressed ones (Paz Gavilán et al., 2006), suggesting more vulnerability of aged-animals to apoptosis.

The ubiquitin-proteasome system (UPS) plays an important role in the cellular processes for protein quality control. UPS maintains the homeostasis of proteins within the cell, ensuring correct protein folding, function, and degradation (Park et al., 2020). The UPS is responsible for the degradation of most short-lived intracellular proteins in eukaryotes by removing over 90% of improperly folded and damaged proteins. Additionally, the UPS actively participates in the regulation of numerous signaling pathways, including mTOR, UPR, as well as both innate and adaptive immune responses. Regulating protein degradation is an integral part of the UPR to alleviate ER stress (Xia et al., 2020). However, this system is impaired with age (Sun-Wang et al., 2020). Age-related UPS dysfunction involves reduced proteasome subunit expression, altered composition, and changes in ubiquitin enzyme activity. Effects may vary by tissue due to differing proteasome activities (Sun-Wang et al., 2020). Proteasome activity remains intact in healthy aged mouse lungs, but caspase-like activity significantly declines compared to young mice (Caniard et al., 2015). Another study confirmed a decline in the proteasome activity in the lungs of 2-year-old aged male rats as compared to that in the lungs of 2-week-old male rats (Keller et al., 2000). When the functions of UPS are compromised, the degradation of ERAD substrates is prevented, causing the accumulation of unfolded or misfolded proteins in the ER lumen. This accumulation triggers the UPR (Ebstein et al., 2019).

In summary, the decline in ER mechanisms for maintaining protein quality and the attenuation of degradation pathways overseeing protein quality control lead to a significant rise in misfolded proteins within the ER, ultimately triggering prolonged ER stress in aging lung tissue.

### 3.3 Hormetic regulation of ER stress on aging

Hormesis, characterized by a dose–response dynamic where low doses stimulate and high doses inhibit the reaction, (Calabrese, 2008), signifies the adaptive responses of cells and organisms to mild or moderate stressors, such as heat, hypoxia, caloric restriction, and oxidative stress. This phenomenon has played a pivotal role in the evolutionary process (Salminen and Kaarniranta, 2010).

ER stress is a paradigm of this hormetic regulation. The buildup of unfolded and incorrectly folded proteins in the ER cavity swiftly initiates ER stress. To overcome this ER stress, cells use a dynamic intracellular UPR process. This mechanism activates adaptive initiatives to adjust and enhance crucial elements of the entire secretory pathway (Hetz and Papa, 2018). If this reaction proves effective, the response restores cellular balance and promotes survival under ER stress. However, persistent ER stress can lead to oxidative stress, inflammation, and apoptosis via UPR pathways (Wang and Kaufman, 2016; Zhang and Kaufman, 2008).

Hormesis of ER stress can also involve the regulation of cell lifespan in a dose-dependent manner. Aging weakens the adaptive UPR-based defense, heightening vulnerability to ER stress and reducing stress resistance. This increases susceptibility to pathological changes, including protein issues, mitochondrial impairments, and disruptions in Ca^2+^ homeostasis, leading to apoptotic cell death.

## 4 ER stress and UPR in different disease models of lung aging

### 4.1 COPD

COPD is marked by partially reversible airflow limitation, chronic inflammation, and emphysematous lung destruction, resembling a condition of accelerated lung aging (Barnes, 2017).

ER stress plays a key role in COPD development, leading to alveolar epithelial cells (AECs) apoptosis (Yu et al., 2022). Elevated GRP78 levels in lung tissues, lavage fluid, and serum are associated with decreased lung function and severe emphysema in smokers and COPD patients (Aksoy et al., 2017; Merali et al., 2014). Cigarette smoke (CS) exposure is the primary driver of COPD pathogenesis and progression (Vogelmeier et al., 2017). CS-induced pathogenic effects involve ER stress and UPR, elevating misfolded protein levels, including impaired PDI, a crucial ER foldase (Kenche et al., 2013; van Rijt et al., 2012). Additionally, IRE1α exacerbates airway inflammation caused by CS through NF-κB signaling (Wang et al., 2017), and it plays a crucial role in nicotine-induced epithelial-mesenchymal transition, contributing to airway remodeling in COPD and impeding cell migration in human bronchial epithelial (HBE) cells (Lin et al., 2022). Cigarette smoke extract (CSE) induces apoptosis by triggering the PERK/eIF2α/CHOP pathway through the superoxide anion (Tagawa et al., 2011).

### 4.2 IPF

IPF, a progressive lung dysfunction, exhibits symptoms like dyspnea and respiratory failure, and its incidence rises with the aging population (Wolters et al., 2014). While the exact pathogenic mechanism is unclear, evidence links ER stress to IPF, observed in lung samples from patients with familial and sporadic IPF (Ghavami et al., 2018; Korfei et al., 2008; Lawson et al., 2008). The profibrotic impacts of ER stress can be communicated across different lung cell types. In AECs of IPF patients, there was a notable rise in the levels of various ER stress markers, including ATF4, ATF6, CHOP BiP, EDEM, and XBP1, particularly observed in type II AECs (Korfei et al., 2008; Lawson et al., 2008). Mice with asbestos-induced lung fibrosis and individuals with asbestosis displayed increased expression of GRP78 in their respective macrophages (Ryan et al., 2014). Additionally, ER stress plays a role in collagen and fibronectin production induced by transforming growth factor β1 (TGFβ1) in fibroblasts (Zimmerman et al., 2013). ER stress has the capacity to influence critical elements of lung fibrosis, including the AECs, polarization of M2 macrophages, and differentiation of myofibroblasts (Burman et al., 2018). CHOP may participate in ER stress-dependent AEC apoptosis (Korfei et al., 2008). In CHOP-deficient mice treated with bleomycin, decreased AEC apoptosis and lung fibrosis were observed (Tanaka et al., 2015). Furthermore, CHOP has been identified as an inducer of M2 polarization in bleomycin-induced pulmonary fibrosis (Yao et al., 2016). The inhibition of ER stress in fibroblasts from patients with IPF significantly reduces TGFβ1-induced myofibroblast differentiation, αSMA expression, and collagen production (Baek et al., 2012). This indicates the potential therapeutic impact of targeting ER stress pathways in pulmonary fibrosis.

### 4.3 Asthma

Asthma, a complex chronic airway disease, results from intricate interactions between genetic, environmental factors, and aging (Nagasaki and Matsumoto, 2013). Elderly patients with asthma are more susceptible to sensitization by allergens than age-matched controls (Burrows et al., 1991). Elderly individuals with asthma experience increased morbidity and mortality along with various comorbidities compared to adults; moreover, it is clinically characterized by neutrophilic inflammation rather than by eosinophilic inflammation and less frequent atopy, with a reduced response to treatment (Boulet, 2016).

The pathogenesis of asthma involves the activation of ER stress and the unfolded protein response (UPR). Genetic factors related to ER stress, such as orosomucoid-like 3 (ORMDL3), contribute to asthma development by influencing calcium homeostasis, ER stress response, and inflammatory processes (Schmiedel et al., 2016; Breslow et al., 2010; Luthers et al., 2020). Previous studies have shown that genetic variation affecting ORMDL3 expression is an important determinant of asthma susceptibility and predisposition to other autoimmune or inflammatory diseases (Moffatt et al., 2007; James et al., 2019). Stimuli such as aeroallergens, microorganisms, smoking, and pollutants are critical factors in the development of allergic asthma; these stimulants can disrupt ER integrity and induce ER stress (Pathinayake et al., 2018; Osorio et al., 2013). According to previous studies, ER stress is activated in AECs and immune cells in the presence of asthma (Pathinayake et al., 2018; Kim and Lee, 2015). The immediate and sustained activation of the UPR signaling pathway initiates allergic airway inflammation (Osorio et al., 2013; Makhija et al., 2014), particularly in severe or asthma unresponsive to steroids through NF-κB modulation (Kim S. R. et al., 2013). Experimental observations in a mice asthma model indicated that the application of chemical chaperones to mitigate the ER stress response displayed promise in alleviating airway hyperresponsiveness (Kim and Lee, 2015; Makhija et al., 2014; Siddesha et al., 2016).

### 4.4 Acute lung injury and ARDS

Elderly individuals show a reduced capacity to resist infections, including influenza (Gross et al., 1995) and COVID-19 (Chen et al., 2021). Thus, elderly persons are more susceptible to acute lung injury (ALI) that culminates into ARDS and show high mortality or prolonged morbidity due to ARDS-related complications and fibrosis (Meduri and Eltorky, 2015). ARDS encompasses a sudden inflammatory reaction triggered by the impairment of the alveolar capillary barrier, stemming from pulmonary or extra-pulmonary origins (Meduri and Eltorky, 2015; Confalonieri et al., 2017), leading to the impairment of pulmonary vascular permeability, alveolar flooding, and diminished respiratory capacity.

Numerous investigations have demonstrated elevated levels of ER stress markers and UPR mediators, including GRP78, in patients with ALI/ARDS and rodent models of lipopolysaccharide-induced ALI (Kim H. J. et al., 2013; Kim et al., 2015; Zeng et al., 2017). Similarly, suppressing ER stress mitigates endotoxin-triggered acute lung injury (ALI) in both *in vivo* and *in vitro* settings. Additionally, restraining the UPR diminishes ALI induced by the Middle East respiratory syndrome coronavirus, acting on the apoptotic pathway downstream of UPR (Sims et al., 2021).

## 5 Mechanism of ER stress and UPR in aging and pulmonary dysfunction

The ER stress signaling components are associated with the cellular signaling network that regulates longevity, thus suggesting a functional relationship between ER stress and aging-related pathways.

### 5.1 ER stress and inflammation

Inflammation is a fundamental protective mechanism of the innate immune system to combat both invading pathogens and endogenously produced toxic substances (Mogensen, 2009), and it is tightly regulated under normal conditions. However, prolonged and sustained inflammation can be detrimental to normal health. Inflammatory pathogenesis is a common mechanism for the development of most age-related diseases (Franceschi and Campisi, 2014). The term inflammaging, a low-grade, chronic, sterile inflammation, is widely accepted as a critical risk factor for aging and major diseases (Santoro et al., 2021). The theory of antagonistic pleiotropy provides a better explanation for the role of inflammation in aging. An evolutionary process, inflammation, manifests significant impacts during early life and adulthood; however, it turns detrimental in old age when the impact of natural selection wanes (Franceschi et al., 2017).

**FIGURE 2:**
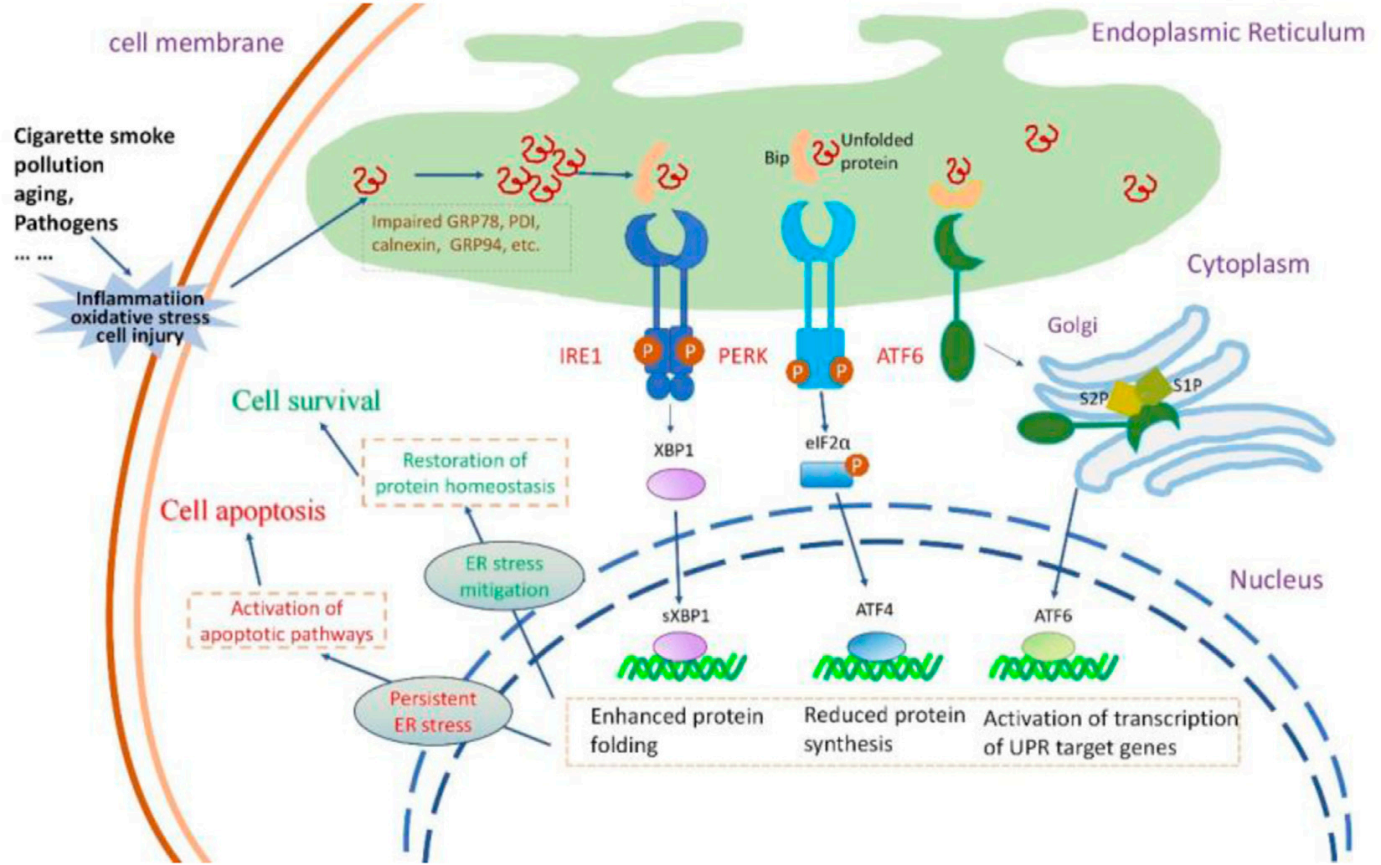
The insults to airway epithelial cells, their impact on UPR and the consequences of UPR activation. Cigarette smoke, pollution, aging, and pathogens invade airway epithelial cells, causing oxidative stress, inflammatory responses, and cellular damage. These insults lead to the accumulation of misfolded or unfolded proteins within the ER lumen.The three signaling pathways of UPR are activated. PERK activation increases the formation of the transcription factor ATF4 that reduces protein synthesis. IRE1 activation results in the production of sXBP1, enhancing the protein folding capacity of the endoplasmic reticulum. Activated ATF6 stimulates the expression of UPR-related genes. Normally, the protective UPR and apoptotic signals are in a balanced state in a cell; however, this equilibrium might incline towards the proapoptotic condition as the damage worsens. If the UPR can successfully alleviate ER stress, the cell can restore protein homeostasis and thus survive. If ER stress persists, the UPR signal can activate the apoptotic pathway, leading to cell death.

The susceptibility of the lungs to aging increases with age, and inflammaging contributes to a proinflammatory environment with diminished resilience against challenges (Faniyi et al., 2022). Inflammation in airway epithelia increases protein synthesis demand, causing an overload of unfolded polypeptide chains in the ER, leading to ER stress and UPR activation (Ribeiro and O'Neal, 2012; Ribeiro and Boucher, 2010) (Figure 2). ER stress also amplifies the cytokine-mediated inflammatory response, leading to the pathogenesis of inflammatory diseases (Zhang and Kaufman, 2008). Thus, a reciprocal relationship appears to be present between ER stress and inflammation; this loop might aggravate the pathological condition of the lung, given that the respiratory tract is an important immune interface with continuous exposure to environmental stress (Schneider et al., 2021).

**FIGURE 3:**
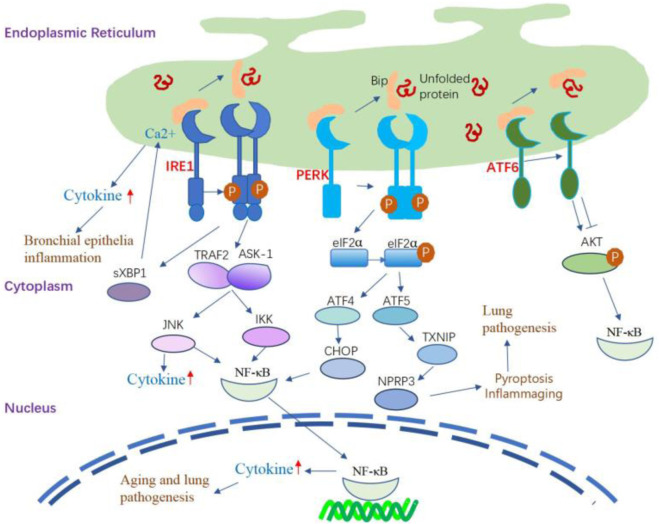
The inflammatory signaling pathway regulated by UPR. IRE1α-dependent sXBP1 activation during the inflammation of human bronchial epithelia can trigger the storage of Ca2+ in the ER and subsequently amplify Ca2+-dependent cytokine generation and release. IRE1α activation leads to the recruitment of TRAF2 and ASK-1, which activate JNK and NF-κB, leading to the production of inflammatory cytokines. The IRE1/TRAF2/ASK1 complex activates inhibitory κ B kinase, which induces the phosphorylation of IκB, leading to the translocation of NF-κB into the nucleus and induction of expression of inflammatory cytokine genes. PERK signaling can activate the signaling of TXNIP, leading to the upregulation of inflammasomes such as NLRP3. PERK modulates NF-κB activation and apoptosis by activating the eIF2α-ATF4-CHOP axis. Additionally, the activation of ATF6 can cause transient phosphorylation of AKT, leading to the activation of NF-κB. ATF6 also positively affects NF-κB activation by inhibiting AKT/GSK3β.

The induction of inflammatory reactions by ER stress involves the activation of all three branches of the UPR. These ER stress sensors activate diverse inflammatory pathways and trigger the expression of inflammatory mediators. IRE1α-dependent sXBP1 activation during the inflammation of human bronchial epithelia can trigger increased storage of Ca^2+^ in the ER (Martino et al., 2009), thereby inducing Ca^2+^-dependent cytokine generation and release (Martino et al., 2009; Ribeiro et al., 2005). In the lung, IRE1α activation is required for the production of cytokines such as IL-8, IL-6, and TNF-α by the inflamed human airway epithelia and human alveolar macrophages (Martino et al., 2009; Hull-Ryde et al., 2021; Lubamba et al., 2015). PERK signaling can activate the signaling of TXNIP, leading to the upregulation of inflammasomes such as NLRP3 (Oslowski et al., 2012). The involvement of NLRP3 activation is crucial in the pathogenesis of several lung diseases, mediating the release of proinflammatory cytokines IL-1β and IL-18, initiating pyroptosis (inflammatory cell death), and playing a key role in the development of inflammaging (Meyers and Zhu, 2020; Liu et al., 2022; Chen et al., 2023). The NF-κB family of transcription factors plays a key role in inflammation and innate immunity, and persistent activation is associated with age-related lung disorders (Korhonen et al., 1997; Zaynagetdinov et al., 2016; Zhang et al., 2013). ER stress serves as the trigger for UPR signaling, intricately linked with the initiation of proinflammatory pathways mediated by NF-κB. The activation of IRE1α recruits TRAF2 and ASK-1, setting off the cascade that leads to the activation of JNK and NF-κB. Simultaneously, PERK plays a pivotal role in influencing NF-κB activation and apoptosis through orchestrating the eIF2α-ATF4-CHOP axis109 (Belali et al., 2022). Furthermore, ATF6 emerges as a positive contributor to NF-κB activation, operating through the mTOR/AKT signaling pathway. This intricate network of interactions emphasizes the complexity of the crosstalk between ER stress, UPR signaling, and the proinflammatory machinery orchestrated by NF-κB (Nakajima et al., 2011) (Figure 3).

### 5.2 ER stress and proteostasis

Proteostasis is a highly conserved biological process and includes an array of mechanisms governing protein functions to maintain cell and tissue homeostasis (Balch et al., 2014). Aging reduces proteostasis, evidenced by declining control over protein quality independent of occurring diseases (Schneider et al., 2021). Persistent biochemical strain in the lungs leads to the buildup of misfolded proteins, detrimental post-translational alterations, and disturbed protein interactions, fostering the development of lung diseases (Balch et al., 2014). Thus, a robust proteostasis network is crucial for lung function and cellular metabolism.

ER stress and UPR are conserved cellular pathways and are important to maintain cellular proteostasis. Dysfunctions of the proteostasis network, particularly disturbances in ER function, are thought to contribute to abnormal protein aggregation (Wang and Kaufman, 2016; Kaushik and Cuervo, 2015). Aging alters ER chaperone and folding enzyme expression, creating an imbalance with heightened ER stress and diminished UPR, leading to chronic ER stress and eventual proteostasis loss (Brown and Naidoo, 2012). This imbalance is also implicated in aging-related disorders through a reduction in global proteostasis at both lung and whole organism levels, which induces senescence in the lungs (Katzen et al., 2022; Wei et al., 2013).

As stated earlier, ER stress was initially associated with IPF when mutations in surfactant protein C (SPC) secreted by type II AECs were identified, leading to misfolding. Type II AECs are secretory cells, and mutations in SPC can further elevate ER stress in these cells (Nakada et al., 2021). In a bleomycin-induced fibrosis mouse model, Increased ER stress triggers the activation of UPR-related receptors in both the entire lung and pulmonary fibroblasts; this led to the proliferation of fibroblasts and excessive collagen deposition (Baek et al., 2012; Hsu et al., 2017). Inhibiting IRE1α was observed to enhance collagen induced by TGFβ1and fibronectin synthesis by fibroblasts in individuals experiencing IPF, along with production of mucus in murine fibrosis models (Ghavami et al., 2018; Chen et al., 2019). Accumulating evidence suggests that damaged proteins are present in the lungs of patients with COPD; enhanced oxidative stress in the lungs, induced by CS, leads to oxidative damage in various lung macromolecules, including proteins, even among individuals who have quit smoking (MacNee, 2005; Pryor and Stone, 1993). CS-induced oxidative load facilitates the buildup of insoluble, polyubiquitinated proteins, observed both in laboratory settings (*in vitro*) and living organisms (*in vivo*) (Kenche et al., 2013; van Rijt et al., 2012). Additionally, the ER-resident foldase PDI experiences oxidation and misfolding due to CS exposure, evident *in vivo* and *in vitro* conditions (Kenche et al., 2013). Hence, CS could compromise ER folding capabilities, resulting in an elevated burden of misfolded proteins within the ER.

Future investigations should focus on specific contributions of UPR pathways to proteostasis in various lung cells and diseases associated with aging.

### 5.3 ER stress and cellular senescence

Cellular senescence is characterized by a persistent cessation of the cell cycle, accompanied by distinctive changes in cellular structure and gene expression. These senescent cells exhibit resistance to programmed cell death through antiapoptotic signaling, maintaining metabolic activity and releasing a combination of inflammatory molecules known as the senescence-associated secretory phenotype (SASP) (Aghali et al., 2022). Cellular senescence exerts a protective effect against cellular stress and tumorigenesis, and it is a crucial biological process frequently observed in embryonic development and wound healing (Storer et al., 2013; Jun and Lau, 2010; Collado and Serrano, 2010). Over time, the buildup of senescent cells becomes a contributing factor to the onset of aging-related diseases (Song et al., 2020; Borghesan et al., 2020), and is considered one of the hallmarks of aging (López-Otín et al., 2013; López-Otín et al., 2023).

Senescent cell accumulation is evident in the lung in response to various stimuli. Lung tissues from individuals with COPD and IPF exhibit senescent cell characteristics, including heightened levels of specific markers (p53, p21CIP1, and p16ink4a) and an upregulated antiapoptotic pathway (Aghali et al., 2022). Moreover, lung homogenates from old mice show a significant increase in SASP components and the inflammatory index compared to those in young mice (Shivshankar et al., 2011).

Senescence, viewed as a stress response to halt the cell cycle of altered cells, is strongly linked with ER stress (Pluquet et al., 2015; Druelle et al., 2016). Research showed that ER stress and UPR is involved in the generation of cellular senescence, and all arms of the UPR may be involved in senescence. For example, PERK activation induces eIF2α phosphorylation and thereafter induce the GADD45α expression (Lee et al., 2019), which is involved in cellular senescence. PERK and IRE1 activation is required to generate SASP components (Matos et al., 2015). The inhibition of IRE1 suppressed H2O2-induced senescence of nucleus pulposus cells (Kang et al., 2022). The ATF6α pathway is involved in cell senescence through its target gene product calreticulin (Shoulders et al., 2013). The secretory state of senescent cells, however, triggers an ER stress response. In senescence, ER and secretory pathway organelles become more active to produce SASP components, which are upregulated and oversecreted. During cellular senescence, changes in morphology occur, marked by expanded and vacuolized cell shapes, along with occasional presence of numerous or enlarged nuclei. The association of vacuolation with uncontrolled activation of UPR has been noted (Peng et al., 2011).

Presently, there is limited direct evidence showing the relationship of ER stress response with cellular senescence in the aging lung. In response to bleomycin injury, IRE1-α signaling has been shown to induce senescence in type II alveolar epithelial cells (AECs), leading to the accumulation of pre-alveolar type 1 transitional cells (PATS) and subsequent fibrosis. Blocking IRE1-α signaling alleviates bleomycin-induced lung fibrosis by reducing senescence and promoting the differentiation of PATS cells to AT1 cells (Auyeung et al., 2022). ER stress may promote cellular senescence in the lung. The lung relies on a strong proteostasis network to handle continuous mechanical stress and various environmental challenges, preventing protein misfolding or recycling damaged proteins. Senescent cells in the lung are most likely to experience ER stress because of the increasing demand for protein folding; moreover, the production of SASP components induces a high burden on the secretory pathway of ER, thereby requiring increased maintenance of proteostasis.

### 5.4 ER stress and stem cells

Adult stem cells play a crucial role in maintaining tissue balance and regenerating cells. Stem cell exhaustion, a decline in both quantity and quality over time, is proposed as a key aging factor (López-Otín et al., 2013; López-Otín et al., 2023).

The intricate structure of the human lung’s epithelium, vital for its functions, depends on the proper functioning of airway epithelial stem cells for maintenance and repair (Basil et al., 2020). The major stem cells in the conducting airways are basal and secretory club cells (Rawlins et al., 2009; Rock et al., 2009). Type II AECs proliferate in the alveoli and generate type I AECs to restore the alveolar epithelium; thus, these cells are considered alveolar stem/progenitor cells (Barkauskas et al., 2013). During the aging process, the number of basal and club cells decrease (Schneider et al., 2021); additionally, airway basal cells exhibit restricted regenerative potential, unable to fully restore a differentiated epithelium, particularly in pathological conditions like COPD (Staudt et al., 2014). With the progress in age, type II AECs remain intact in number but exhibit a decline in their self-renewal and differentiation capacity (Schneider et al., 2021).

Stress affects stem cells, especially hematopoietic stem cells (HSCs) in the physiological hierarchy. The UPR in HSCs regulates self-renewal, resolving stress or initiating apoptosis (Kharabi Masouleh et al., 2015). UFBP1 deficiency elevates ER stress and UPR activation, causing the death of hematopoietic stem/progenitor cells (van Galen et al., 2014). UFBP1 plays various roles in hematopoietic cell survival and differentiation by modulating ER homeostasis and regulating gene expression specific to the erythroid lineage (Cai et al., 2015). The targeted removal of the vital chaperone GRP78/BiP from type II AECs in the lung led to a decline in the regenerative ability of type II AECs. This deletion also triggered activated TGF-β signaling, contributing to spontaneous age-dependent fibrotic lung remodeling characterized by interstitial pneumonia-like features. An ER stress inhibitor alleviated fibrosis in mice lacking Grp78 in epithelial cells and IPF lung slices, highlighting a potential aging-IPF connection through ER stress (Borok et al., 2020). An *in vitro* study in type II AECs revealed that CSE could activate ER stress-induced apoptosis by activating JNK and caspase-12 in a dose- and time-dependent manner; however, this activation of the ER stress signaling pathway can also initiate a protective mechanism by inducing HRD1, which is a specific ubiquitin E3 ligase on the ER membrane, to counteract CSE-induced UPR for protecting type II AECs (Tan et al., 2017).

The mitochondrial UPR (UPRmt), a regulatory mechanism for preserving adult stem cells, is conserved across tissues. Dysregulation of this protective mechanism contributes to the functional decline of stem cells and tissue degeneration over time (Mohrin et al., 2018). Alternatively, the constant UPRmt activation, along with tissue-specific HSP60 loss, can detrimentally impact stemness (Berger et al., 2016). In AECs of the lung, by inducing ATF4-dependent UPRmt and mitochondrial dysfunction, ER stress is implicated in the development of diverse pulmonary disorders, and type II AECs may play a role in this process (Jiang et al., 2020).

### 5.5 ER stress and mitochondria

Mitochondria, essential cellular organelles, produce energy and reactive oxygen species (ROS), influencing physiological functions, while also playing a crucial role in governing inflammatory responses, innate immunity, cell death, and aging (Schafer et al., 2017; Miwa et al., 2022). As the cell ages, its mitochondria accumulate abnormalities, but increased mutations in mtDNA, thereby leading to declined mitochondrial function (Mora et al., 2017). Mitochondrial dysfunction contributes to impaired lung functions; affects the trachea, bronchi, bronchioles, alveoli, and interstitium; hinders lung recovery after an injury (Caldeira et al., 2021).

**FIGURE 4:**
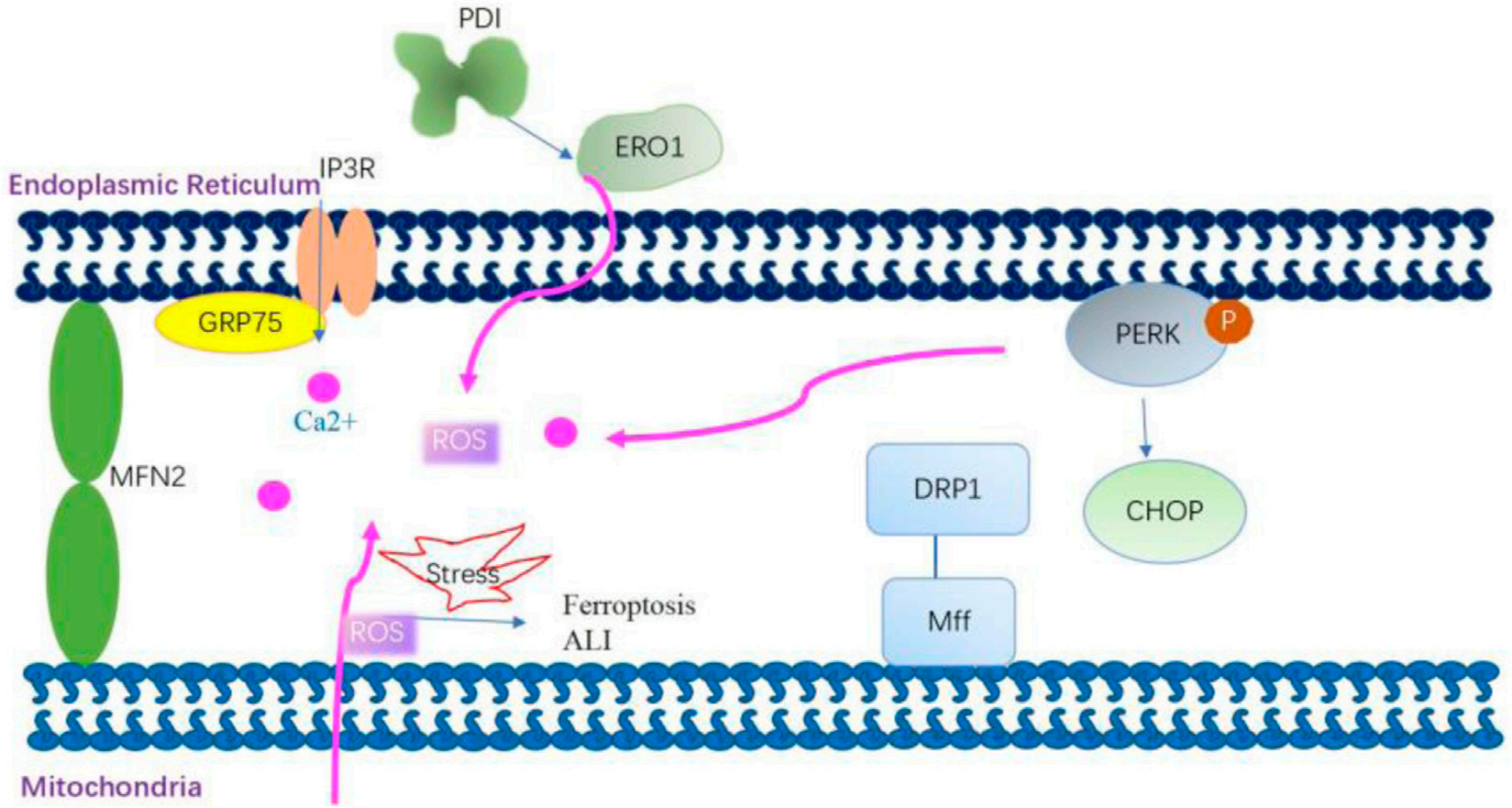
Mitochondria-associated membrane modulates mitochondrial Ca^2+^ uptake and improves mitochondrial dynamics and redox balance. Grp75 interacts with transglutaminase type 2 in mitochondria-associated membranes (MAMs) and then stabilizes the interaction between Grp75 and IP3R to enhance ER–mitochondria association. The enhanced ER–mitochondria association promotes the ER-mitochondrial Ca2+ flux. Dynamic-related protein 1 (Drp1) is recruited to the outer mitochondrial membrane by the fission receptor mitochondrial fission factor, which increases ER–mitochondria interactions by promoting the formation of tubules in the ER. Mitofusin 2 (Mfn2) on the ER interacts with Mfn2 on the mitochondria to form homo-complexes. These homo-complexes can tether the ER and mitochondria to enhance MAM formation. PERK is also a crucial component of MAMs. PERK plays a role in establishing a physical and functional association between the ER and mitochondria. The knockout of the PERK gene affects the ER–mitochondria association, thereby disturbing ER morphology, decreasing Ca2+ transfer from the ER to the mitochondria, and reducing the transmission of ROS signaling from the ER to the mitochondria. mtROS-mediated MAM dysfunction is involved in the mechanism of arsenic-induced ferroptosis and ALI.

The ER serves as a central hub establishing physical connections with mitochondria. MAMs are specialized subdomains facilitating close proximity between the ER and mitochondria, fostering intricate cross-talk (Phillips and Voeltz, 2016) (Figure 4). This juxtaposition enhances mitochondrial Ca^2+^ uptake and improves mitochondrial dynamics and redox balance; it also triggers apoptosis under excessive ER stress condition (van Vliet and Agostinis, 2018). Various mitochondrial and ER stress-associated proteins such as Drp1, Mfn2, and PERK are involved in the structure and functions of MAMs (Mao et al., 2022). Disturbance in the MAM during ER stress is implicated in the aging process and age-related diseases (Moltedo et al., 2019; Volgyi et al., 2015). In an *in vitro* study using HBE cells, ER stress induced by titanium dioxide nanoparticles mediated autophagic cell death through MAM disruption (Yu et al., 2015). The exposure of human airway smooth muscle (hASM) cells to the proinflammatory cytokine TNF-α triggers ER stress pathways, leading to the disruption of ER–mitochondria interaction, inhibition of Mfn2 expression, and impairment of mitochondrial mobility (Yap et al., 2020; Delmotte et al., 2017).

In aged mice and IPF patients, ATF4 and UPRmt were induced in lung alveolar epithelial cells. Inducing ATF4 in mouse AECs worsened pulmonary UPRmt, inflammation, and death post-bleomycin injury (Jiang et al., 2020). Thus, it can be inferred that the UPRmt generally slows mitochondrial activity for organelle recovery but may raise the risk of declining oxidative phosphorylation, membrane potential loss, and increased ROS production in AECs.

### 5.6 ER stress and oxidative stress

ROS, which are continuously produced in all aerobic organisms, are derived from oxygen metabolism as byproducts of cell respiration (Lenaz, 2012) and include superoxide anions (O2·-), hydroxyl radicals (·OH), and hydrogen peroxide (H2O2). Oxidative stress arises due to an imbalance between the production of ROS and antioxidant mechanisms. The causal relationship among ROS, aging, age-related pathologies, and senescence has been studied intensively based on the free radical theory of aging (Harman, 1956) and the mitochondrial theory of aging (Alexeyev, 2009). A recent study investigated whether ROS is beneficial or detrimental to extend lifespan; this is because the basal level of ROS is essential to regulate biological processes and metabolic homeostasis (Zhang et al., 2019). The hormetic response of ROS is not advantageous for the mammalian lung. Pulmonary disorders linked to oxidative stress involve increased ROS production and decreased antioxidants. Asthma patients, for instance, show elevated ROS in macrophages and diminished lung antioxidants (Comhair and Erzurum, 2010). Exposure to oxidative toxicants in the lungs causes tissue damage, inflammation, and the accumulation of phagocytic leukocytes, further enhancing oxidant production (van der Toorn et al., 2009).

Prior research indicates a close interconnection between ER stress and oxidative stress. These two stressors mutually reinforce each other, playing roles in several chronic conditions associated with aging (Kim et al., 2008; Lee and Lee, 2022). The ER serves as an optimal setting for protein oxidation and folding. Oxygen is essential for the redox coupling between ER oxidoreductase 1 (ERO1) and the final enzyme PDI, enabling the formation of protein disulfide bonds unique to the ER (Cao and Kaufman, 2014). This ER redox system converts oxygen to H2O2, thereby contributing to the total cellular ROS, although the amount of ROS thus generated is very small as compared to that produced in the mitochondria (Tu and Weissman, 2004). During ER stress, ROS production is elevated through NADPH oxidases, mainly through Nox2 and Nox4 (Santos et al., 2014). Moreover, a substantial quantity of activated JNK interacts with the MAM-linking protein Sab, leading to the release of ROS (Win et al., 2014). Global transcriptome studies revealed that ER stress did not alter the expression of UPR-associated genes in aging lungs (Misra et al., 2007); however, the expression of Grp78 was decreased, and the expression of Grp94 and proapoptotic CHOP was increased in type II AECs (Borok et al., 2020). CHOP is involved in excessive ROS production by increased expression of ERO1 (Marciniak et al., 2004). Oxidative stress enhances protein oxidation, disturbs ER protein folding, and leads to the buildup of misfolded oxidized protein substrates. This is exacerbated by the compromised function of the chaperone system and other cellular “quality control” mechanisms due to proteins modified by ROS (Muller et al., 2013). Consequently, the simultaneous occurrence of oxidative stress and ER stress constitutes the initial response to pathological challenges. The inhibition of these cellular stress factors can stop the formation of a vicious loop and thus prevent pathological developments in the lung. Moreover, it possesses the ability to protect against acute lung injury (ALI) induced by sepsis. This protection is achieved by suppressing ER stress and mitochondrial dysfunction mediated by oxidative stress through the activation of the SIRT1/AMPK pathways specifically within the lung (Sang et al., 2022).

### 5.7 ER stress and genome integrity

Genome integrity is constantly threatened by damage due to exogenous agents and intrinsic biological processes that can induce DNA damage (Lindahl and Barnes, 2000). A common phenomenon in the aging process is the accumulation of genetic damage throughout the life (López-Otín et al., 2013; López-Otín et al., 2023). Accordingly, cells have evolved an intricate and specific DNA damage response (DDR) cascade responsible for the recognition and repair process (Sancar et al., 2004). The DDR cascade mainly involves the phosphoinositide 3-kinase proteins (PI3Ks) (ATM, ATR, and DNA-PK) and poly (ADP‐ribose) polymerase (PARP).

DNA damage, which is mostly oxidative in nature, plays a key role in the development of aging-related chronic diseases. In asthma, exposure to environmental allergens induces oxidative DNA damage in airway epithelial cells, with the repair of this damage linked to Th2 cytokine secretion and the initiation of allergic inflammation (Zahiruddin et al., 2018).

UPR and DDR are the two important mechanisms to maintain cell homeostasis. Although these adaptive mechanisms occur in the ER and nucleus, respectively, they are involved in the mechanisms and signaling pathways that modulate genome integrity. The DDR-related protein ATM might be associated with the overlap between the DDR and ER. As discussed earlier, oxidative stress is a natural biological process that occurs in all cells and can also occur in aging and aging-related lung disorders. The mutated versions of ATM exhibit resistance to oxidative stress and a minimal impact on the DNA damage response (DDR). Yet, they assist in the removal of toxic protein buildup (Lee et al., 2018). Thus, ATM may not only act as an oxidative stress sensor but can also sense alterations in other cellular compartments, including the ER (Hotokezaka et al., 2020; He et al., 2009). The nonhomologous DNA end-joining double-strand break (DSB) repair pathway in *Saccharomyces cerevisiae* is influenced by the exogenous expression of mammalian XBP1, which regulates H4 acetylation (Tao et al., 2011). Additionally, sXBP1, under ER stress, controls the transcription of a cluster of DNA repair genes (Acosta-Alvear et al., 2007). SRC is a protein tyrosine kinase and can be activated by the UPR; following its activation, it creates a complex with IRE1α, leading to the relocation of ER chaperones to the cell surface (Tsai et al., 2018). SRC is also involved in the regulation of the DDR by terminating the ATR-Chk1-dependent G2 DNA damage checkpoint (Fukumoto et al., 2014).

To date, few studies have provided evidence of an association between DNA damage and ER stress in the lungs. The obtained results indicate that the DDR and UPR can direct a coordinated response to manage cellular stress, which may be relevant in aging and lung diseases. Additional experimental evidence is needed to understand the interaction between this UPR branch and the cellular machinery involved in genomic stability, particularly pertaining to aging and related lung diseases.

### 5.8 ER stress and autophagy

Autophagy is a conserved multistep catabolic process that degrades unfolded proteins, cytoplasmic contents, and damaged organelles by forming autophagosomes. It is currently viewed as an association between metabolic and proteostatic signaling that can determine key physiological decisions ranging from cell fate to organismal lifespan (Kaushik et al., 2021).

**FIGURE 5:**
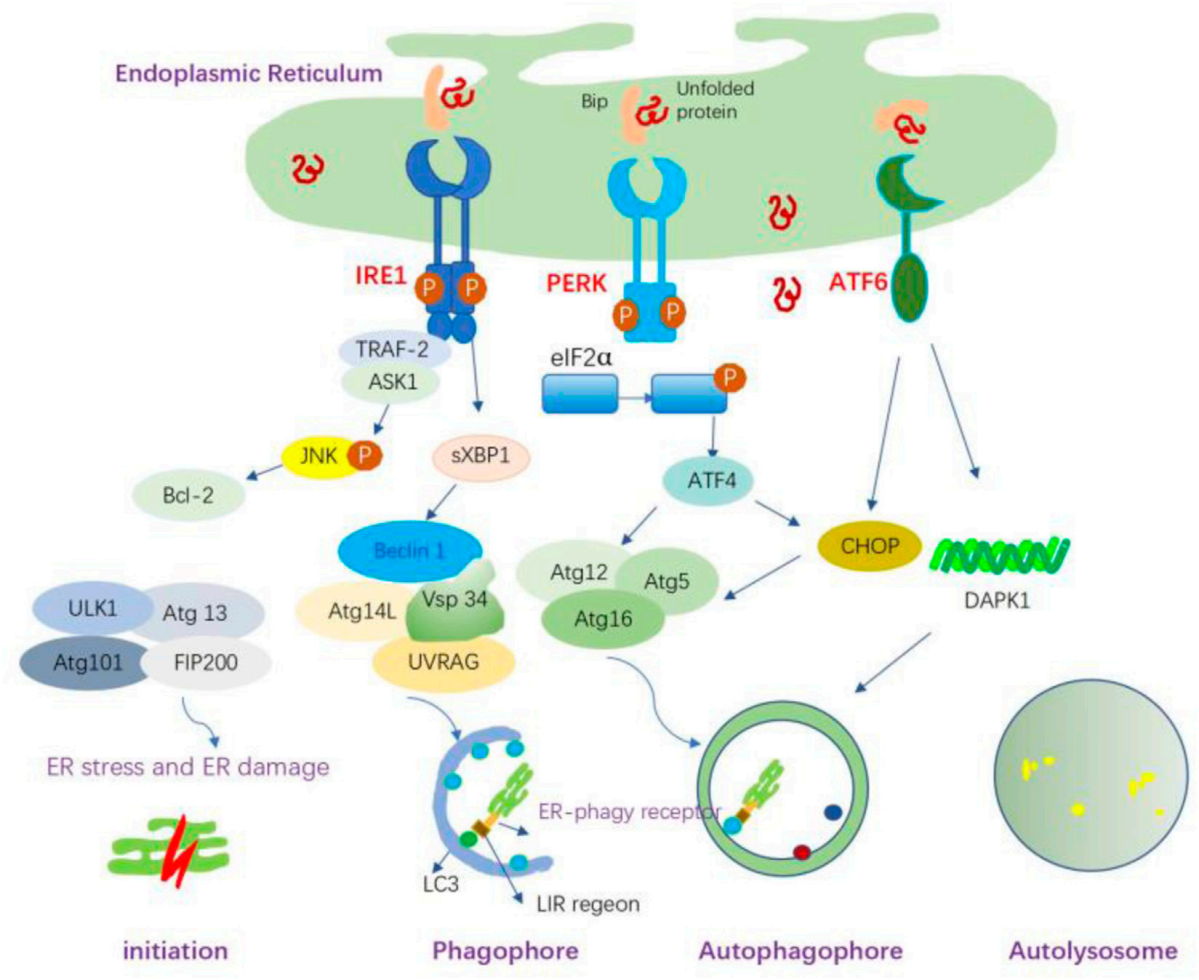
ER stress-mediated autophagy and the possible mechanisms. ER stress can induce autophagy through the IRE1α, PERK, and ATF6 signaling pathways. IRE1 forms a complex with TRAF-2, which subsequently binds to ASK1, leading to the phosphorylation of JNK and the promotion of Bcl-2 activation, thereby activating autophagy. sXBP1 also triggers transcriptional activation of Beclin-1. The activated PERK induces autophagy through ATF4-driven transcriptional regulation of Atg12, whereas ATF4-mediated CHOP activation transcriptionally induces Atg5 expression. Atg5, Atg12, and Atg16L form the Atg5-Atg12-Atg16L complex, which is involved in the elongation process. ATF6 indirectly regulates autophagy through CHOP; it upregulates the expression of DAPK1, which phosphorylates Beclin-1 and mediates autophagy initiation. ER-phagy is initiated to remove damaged ER organelles. ER-phagy receptors on the ER membrane mediate the interaction between LC3 and its target ER sites through the LC3-interacting region (LIR).

ER stress and autophagy are two closely interconnected response pathways to cellular stress (Senft and Ronai, 2015; Bhardwaj et al., 2020). In eukaryotic cells, ER stress can activate lysosomes through the p38 MAPK pathway, thereby activating Chaperone-Mediated Autophagy (CMA) (Li et al., 2017). Autophagy degrades unfolded proteins when the canonical, proteasome-dependent pathway is impaired or overwhelmed by an excessive amount of ubiquitinated proteins. Therefore, autophagy is considered the last approach to restore the homeostasis of the ER. Autophagy can be triggered following UPR-inducing stimuli (Nijholt et al., 2011) and serves as a pro-survival mechanism under ER stress conditions (Figure 5). However, ER stress can also disrupt lysosomal homeostasis, leading to an obstruction of autophagic flux. For instance, the level of serum lysosomal-associated membrane protein 1 (LAMP1) was significantly decreased in pre-eclampsia patients compared to normal pregnant women, suggesting potential lysosomal dysfunction due to ER stress in pre-eclamptic placentas (Nakashima et al., 2019).

Beyond autophagy, the interplay between the ER and lysosomes manifests in various other ways. Research has demonstrated that SNX19 restricts endolysosomal motility via its interaction with the endoplasmic reticulum, a process crucial for the concentration of endolysosomes in the perinuclear region (Saric et al., 2021). These interactions not only contribute to the stability of the intracellular environment but also play a pivotal role in a range of pathological conditions, including neurodegenerative diseases, metabolic disorders, and cancer.

ER stress and UPR pathways share mechanisms that control autophagy, collectively influencing responses to diverse stimuli. This interplay is implicated in the development of lung diseases. For example, during respiratory viral infection, the deficiency of the autophagy protein Map1-LC3b can increase ER stress and associated IL-1 secretion and lead to IL-17-dependent lung pathology (Reed et al., 2015). CS induces the expression of lysosome-associated membrane protein 2A (LAMP2A) and chaperone-mediated autophagy (CMA) in an Nrf2-dependent manner in HBE cells. The suppression of LAMP2A and CMA inhibition enhances UPR and apoptosis; this effect can be reversed by the induction of LAMP2A expression (Hosaka et al., 2020). Thus, careful manipulation of autophagy in the context of UPR may enable to develop new therapeutic strategies for aging-related lung diseases.

### 5.9 Sirtuins exert an antiaging effect by regulating ER stress

Sirtuins, the family of NAD + -dependent protein deacetylases, have been studied extensively as a potential antiaging factor (Bonkowski and Sinclair, 2016). Decreased sirtuin activity, leading to elevated histone acetylation, causes aging phenotype in certain organisms (Wątroba et al., 2017).

Initially recognized for their deacetylase function, sirtuins are now recognized for their involvement in various biological activities and are a focal point in research on ER stress and the UPR. Sirtuins deacetylate sXBP1, potentially preventing ER stress-mediated release of proinflammatory cytokines and apoptosis (Wang et al., 2011). XBP1 induces SIRT7 expression, reducing ER stress-related protein expression (Shin et al., 2013). SIRT1 plays a crucial role in protecting against ER stress-related lung damage, including in sepsis-induced lung injury and hyperoxic ALI (Wang et al., 2019; Sun et al., 2017). Lower serum SIRT1 levels are observed in COPD patients, while SIRT1 upregulation protects against COPD by reducing apoptosis and ER stress (He et al., 2019). SIRT1 elevation promotes autophagy, but using SIRT1 inhibitors reverses this effect, worsening COPD by intensifying ER stress (Tang and Ling, 2019).

## 6 Targeting ER stress and UPR in aging and aging-related pulmonary diseases

Given the significance of ER stress and the UPR signaling pathway in the process of aging and the pathogenesis of pulmonary diseases, strategies to target these two factors are crucial for the intervention of lung diseases. As an alternative strategy, the activation of the adaptive UPR or the suppression of the maladaptive UPR by using appropriate compounds has considerable potential.

### 6.1 Pharmacological intervention

#### 6.1.1 Metformin

Metformin, a pharmaceutical agent commonly prescribed for the management of type 2 diabetes mellitus, demonstrates additional therapeutic benefits beyond glycemic control. In mouse lungs, it mitigates cigarette smoke-induced emphysematous COPD pathologies through the AMPK pathway, concurrently suppressing the unfolded protein response (UPR) and endoplasmic reticulum (ER) stress (Polverino et al., 2021).

#### 6.1.2 Melatonin

Melatonin, a neuroendocrine hormone primarily produced in the pineal gland, serves various biological roles, including sleep modulation, circadian rhythm regulation, immune system enhancement, antioxidant properties, antiaging influence, and antitumor activity (Luo et al., 2020). Additionally, it can impede the initiation of NLRP3 inflammation and ER stress in COPD (Mahalanobish et al., 2020).

#### 6.1.3 Resveratrol

Resveratrol is a natural phenolic compound found in many foods. Many studies have highlighted its role in longevity and prevention of aging-related disorders (Harikumar and Aggarwal, 2008). Resveratrol diminishes inflammation and apoptosis by easing ER stress via the Akt/mTOR pathway in cases of allergic airway inflammation induced by fungi. This provides potential therapeutic approaches for lung diseases triggered by allergic reactions to fungi (Wang S. et al., 2022).

#### 6.1.4 SIRT1

SIRT1 attenuates ER stress and apoptosis in the rat models of COPD and septic associated-lung injury (Zhang L. et al., 2020; Wang F. et al., 2022). Interestingly, melatonin protects against COPD through the attenuation of apoptosis and ER stress by upregulating SIRT1 expression in rats (He et al., 2019).

**FIGURE 6:**
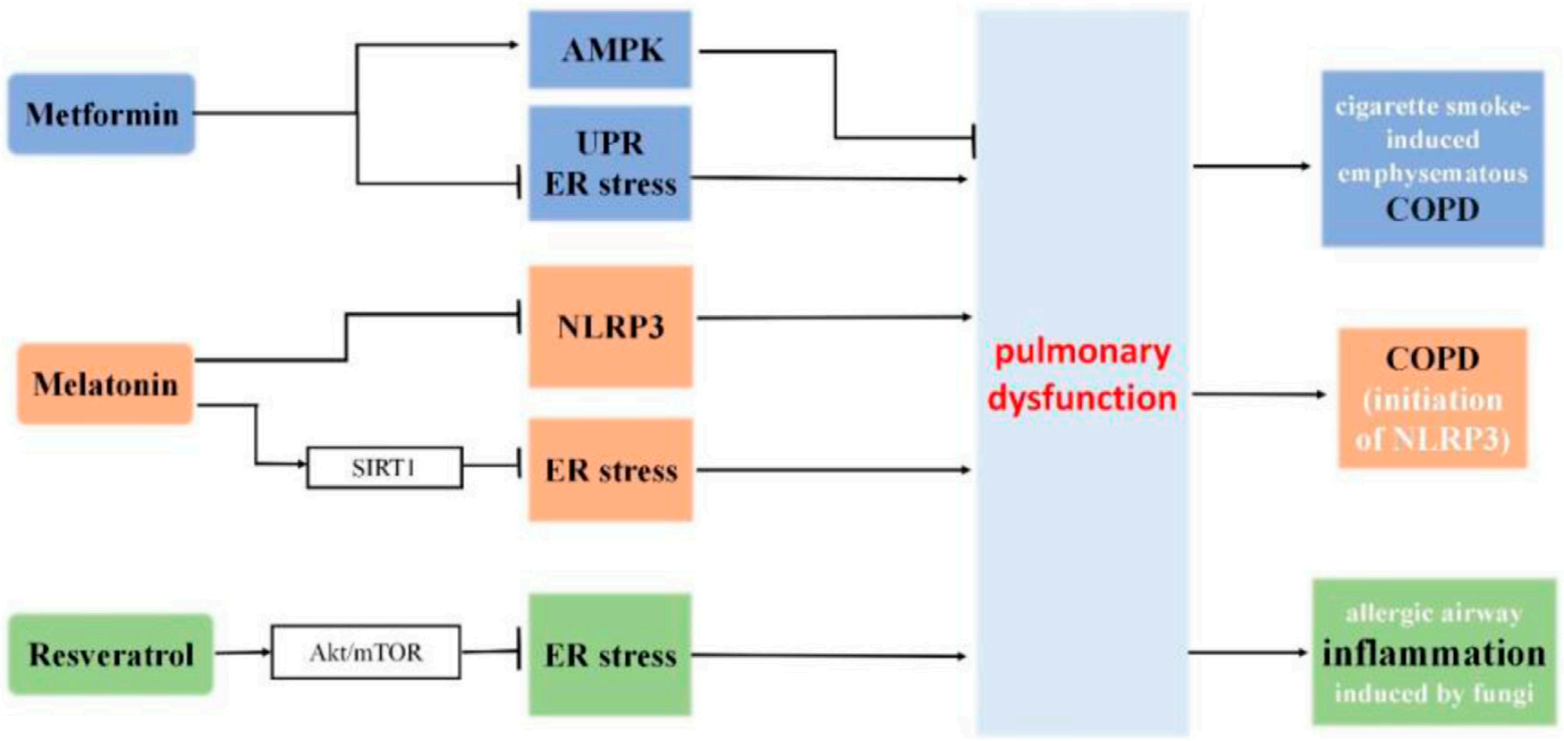
The roles of metformin, melatonin and resveratrol in ER stress and pulmonary dysfunction. Metformin mitigates cigarette smoke-induced emphysematous COPD pathologies through the AMPK pathway, concurrently suppressing the unfolded protein response (UPR) and endoplasmic reticulum (ER) stress. Melatonin protects against COPD through the attenuation of apoptosis and ER stress by upregulating SIRT1 expression in rats. Additionally, it can impede the initiation of NLRP3 inflammation and ER stress in COPD. Resveratrol diminishes inflammation and apoptosis by easing ER stress via the Akt/mTOR pathway in cases of allergic airway inflammation induced by fungi.

While compounds directed at ER stress or UPR elements exhibit promise in averting and treating age-related lung diseases, clinical trials are necessary to comprehensively assess their impact on ER stress and UPR (Figure 6).

### 6.2 Lifestyle adjustments

Regular exercise is known to promote healthy aging and mitigate aging‐related diseases (Booth et al., 2012; Viña et al., 2016). Exercise also shows protective effects against COPD (Rochester et al., 2015), IPF, and COVID-19 (Dwyer et al., 2020; Yu et al., 2019). The mechanisms underlying the effects of exercise on longevity are yet to be fully clarified; however, regular exercise can reverse ER dysfunctions (Estébanez et al., 2018).

In obese individuals, physical exercise alleviates ER stress by reducing the expression and release of the GRP78 chaperone (Khadir et al., 2016). Exercise influences the adaptive Unfolded Protein Response (UPR) for the purpose of muscle remodeling, as evidenced by the analysis of biopsy samples from human skeletal muscle. Nevertheless, this capability is compromised with the progression of aging (Khadir et al., 2016).

## 7 Future perspectives and conclusion

Based on the findings and hypotheses presented in this review, ER homeostasis, ER stress, and UPR have been extensively implicated in aging and aging-related lung disorders. Regulating ER stress shows potential as a strategy to mitigate age-related health decline and avert lung disorders.

Aging itself is, however, a highly complicated process, and ER stress has a complex role in the biology of aging. Investigations on the role of ER stress in aging and aging-related lung diseases thus remain a challenge. The bulk of research on ER stress relies on simplistic models or undifferentiated cells, potentially limiting its applicability to understanding the pathophysiology of well-differentiated pulmonary cell types, especially in the context of aging. The efficacy of manipulating ER stress could be contingent on its collective influence on each of these cellular categories. Therefore, more detailed mechanistic studies are required on the interaction between ER stress and disease progression in the aging lung and on the modulation of this association for clinical translation. Nonetheless, the investigations reviewed here provide substantial insights into the role of ER stress and UPR in aging-related lung diseases, and these insights could prove to be valuable for developing effective therapeutic prevention and intervention strategies.
